# A patient with bilateral pheochromocytoma as part of a Von Hippel-Lindau (VHL) syndrome type 2C

**DOI:** 10.1186/1477-7819-5-112

**Published:** 2007-10-08

**Authors:** Jennifer MJ Schreinemakers, Bernard A Zonnenberg, Jo WM Höppener, Frederik J Hes, Inne HM Borel Rinkes, Cornelis JM Lips

**Affiliations:** 1 Dept. of Surgery, University Medical Center Utrecht, Heidelberglaan 100, 3584CX Utrecht, The Netherlands; 2 Dept. of Internal Medicine, University Medical Center Utrecht, Heidelberglaan 100, 3584CX Utrecht, The Netherlands; 3 Dept. of Endocrinology and Metabolism, University Medical Center Utrecht, Heidelberglaan 100, 3584CX Utrecht, The Netherlands; 4 Leiden University Medical center, Center for Human and Clinical Genetics, The Netherlands

## Abstract

**Background:**

Von Hippel-Lindau (VHL) disease is an autosomal dominant inherited disease. It is relatively recent that type 2C was identified as a separate group solely presenting with pheochromocytomas. As an illustration, an interesting case is presented of a pregnant woman with refractory hypertension. It proved to be the first manifestation of bilateral pheochromocytomas. The family history may indicate the diagnosis, but only identification of a germ line mutation in the DNA of a patient will confirm carriership.

**Case presentation:**

A 27 year pregnant patient with intra uterine growth retardation presented with hypertension and pre-eclampsia. Magnetic resonance imaging revealed bilateral adrenal pheochromocytoma. She underwent laparoscopic adrenelectomy and a missense mutation (Gly93Ser) in exon 1 of the VHL gene on chromosome 3 (p25 – p26) was shown in the patient, her father and her daughter confirming the diagnosis of VHL.

**Conclusion:**

In almost all VHL families molecular genetic analysis of DNA will demonstrate an inherited mutation. Because of the involvement in several organs, periodic clinical evaluation should take place in a well coordinated, multidisciplinary setting. VHL disease can be classified into several subtypes. VHL type 2C patients present with pheochromocytomas without evidence of haemangioblastomas in the central nervous system and/or retina and a low risk of renal cell carcinoma. Therefore, in such families, periodic clinical screening can be focussed on pheochromocytomas.

## Background

A pheochromocytoma is a common tumour in patients with von Hippel-Lindau disease (VHL disease). VHL disease is an autosomal, dominant inherited tumour syndrome with an estimated prevalence of 2–3 patients per 100,000 persons. VHL is characterised by highly vascularised tumours in different organs.

Here, we describe a patient with VHL type 2C who was referred to our hospital with bilateral pheochromocytomas during pregnancy. DNA-analysis of the VHL-gene revealed that several family members had the same mutation in the VHL gene.

## Case presentation

A 27 year old pregnant woman, the index patient, was referred to the obstetric department of our University Medical Centre with hypertension and pre-eclampsia at 26 weeks. Ultrasonography showed intra-uterine growth retardation. History showed that the patient's father had undergone a resection of the right adrenal gland at the age of 48 because of a pheochromocytoma. Clinical evaluation of the index patient with magnetic resonance imaging (MRI) revealed bilateral adrenal pheochromocytomas, the right tumour being 4 centimetres and the left 1 centimetre in diameter. Twenty-four hours urine analysis showed a creatinin of 8.8 mmol/24 hours (reference 9–18 mmol/24 hours), metanephrines 21.6 μmol/24 hours (ref:<1–8 μmol/24 hours), VMA 64 μmol/24 hours (8–36 μmol/24 hours), free adrenaline 146 nmol/24 hours, free noradrenaline 4850 nmol/24 hours (90–470 nmol/24 hours) and free dopamine 4450 nmol/24 hours (420–2600 nmol/24 hours). She had no *café-au-lait *spots. She received alpha-adrenergic receptor blockade (doxazosine 160 mg), calcium channel blockers (nifedipine 30 mg) and beta-receptor blockade (metoprolol 100 mg) one week later. At 28 weeks a Caesarean section was performed due to pre-eclampsia and a daughter was born weighing 745 grams with a length of 33 cm. Her head circumference measured 23 cm. During this procedure the right adrenal gland was removed through a subcostal incision without any intra-operative complications.

The obstetrician referred the mother to the endocrinologist. Additional family history demonstrated that the patient's paternal grandmother had died at the age of 42 as a result of a stroke. It remains unclear whether she suffered from a pheochromocytoma. The index patient's grandmother's brother also died from of a stroke at the age of 57. Both patients may have had a pheochromocytoma since cerebrovascular accidents are known complications. Another theory is they both suffered from cerebellar haemangioblastomas. However, these cerebellar neoplasms more often present with cerebellar signs and symptoms as headache, nausea and vomiting, dizziness, blurred vision, nystagmus, ataxia, intention tremors, coordination deficits and at a later stage herniation of the cerebellum, oedema and death. This suggests that pheochromocytomas would be the more plausible explanation.

Three months later the patient underwent an uncomplicated laparoscopic adrenalectomy for the left pheochromocytoma. Subsequently, she was treated adequately with hydrocortisone, mineralocorticoids and dehydroepiandrosterone.

The differential diagnosis for the index patient focussed on VHL disease and multiple endocrine neoplasia type 2. DNA analysis, did not demonstrate a RET proto-oncogene mutation. Later, a missense mutation (Gly93Ser) in exon 1 of the VHL gene on chromosome 3 (p25 – p26) was shown in the patient, her father and her daughter. The diagnosis of VHL disease was now proven based on the positive family history for pheochromocytoma and the mutation in the VHL gene. Further diagnostic work-up revealed two small lesions suspicious for inactive angiomas on the right retina in the index patient and the left without any abnormalities.

The family had many questions concerning the risks of the daughter having VHL disease and was referred to the Department of Clinical Genetics for counselling.

Additional ultrasonography in the father showed epididymal cysts on the left side and bilateral renal cysts. An MRI of the brain and spinal cord showed no signs of haemangioblastomas and examination of the eyes no retinal haemangioblastomas.

**Figure 1:**
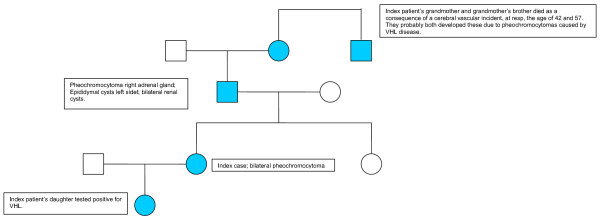
pedigree of the family.

The family tree of the family is depicted in Figure 1. The sister of our index patient did not inherit the family's mutation. The patient, her father, and daughter remain in a periodical screening program for VHL disease for early detection of other VHL associated tumours, but especially pheochromocytomas.

## Discussion

About 12–24% of all pheochromocytomas are hereditary and occur as a feature of familial syndromes  [1-8]. Bilateral adrenal pheochromocytomas are often part of a familial tumour syndrome most likely being multiple endocrine neoplasia syndrome type 2 (MEN 2) or von Hippel-Lindau syndrome (VHL). Bilateral extra-adrenal pheochromocytomas often occur in paraganglioma (PGL) syndromes[9]. In neurofibromatosis type 1 (NF1) adrenal pheochromocytomas occur in nearly 1% and bilateral localization is an exception.

In patients with at least one typical VHL-associated tumour, a clinical diagnosis of VHL disease can be made if a positive family history of a typical VHL-associated tumour exists which was the case in the reported family.

Typical tumours in VHL disease are haemangioblastomas of the retina, cerebellum and medulla, renal cell carcinomas (RCC), and pheochromocytomas. Other VHL tumours are cysts with adenomas in the kidneys, pancreatic islets, epididymis, ligamentum latum, fallopian tubes and ovaries, and endolymphatic sac tumours [10,11]. A wide range of disease and age manifestations exists between families and within families[11,12].

VHL disease is classified into four subtypes, type 1 without pheochromocytoma, and type 2A, B and C having risk of development of pheochromocytoma. Patients with type 2A have a low risk of RCC while type 2B patients have a high risk of RCC. VHL type 2C confers an increased risk of pheochromocytomas without other manifestations of the disease. In type 1 families deletions in the VHL gene are often detected, whereas in type 2 disease missense mutations are most often encountered. It is presumed that the genotype-phenotype correlation in VHL disease is a reflection of the altered pVHL function caused by a mutation. Genotype-phenotype correlation for VHL disease and possible responsible pathophysiological mechanisms are shown in Table 1[10,11,13-15].

**Table 1:** Genotype-phenotype correlation for VHL disease and possible responsible pathofysiological mechanisms.

Type VHL	Type of VHL gene germline mutation	Retinal HAB	CNS HAB	RCC	PHEO	Mechanisms for VHL mediated tumorigenesis
1	Missense	+	+	+	-	Loss of function (i.e. HIF decreased)
	Microdeletions					
	Insertions					
	Splice site					
	Nonsense					
	Large deletions					
2A	Missense	+	+	-	+	Loss of function (HAB) (i.e. HIF decreased)
						Gain of function (PHEO) Fibronectin binding decreased
2B	Missense	+	+	+	+	Loss of function (HAB + RCC) (i.e. HIF decreased)
						Gain of function (PHEO) Fibronectin binding decreased
2C	Missense	-	-	-	+	Gain of function (PHEO) Fibronectin binding decreased

VHL: Von Hippel-Lindau, HAB: haemangioblastoma, CNS: central nervous system, RCC: renal cell carcinoma, PHEO: pheochromocytoma, HIF: hypoxia-inducible factor; +: tumour present, -: no tumour

This family appeared to have only pheochromocytomas as part of the VHL syndrome and has therefore been classified as VHL type 2C. Renal cysts and epididymal cysts often occur in the normal population and can not be regarded as pathognomonical for VHL disease.

### Molecular genetics

Carriership of a germline mutation in the Von Hippel-Lindau gene predisposes to the VHL-disease. The VHL gene is a relatively small gene of approximately 14.500 basepairs (bp) of genomic DNA. It is a tumour suppressor gene and a tumour cell develops when inactivation of both copies of the VHL gene occurs.

The normal VHL gene produces two proteins with similar biochemical and functional behaviour, both are addressed as pVHL. Depending on the specific tissue, they are functioning as co-activator or co-repressor. The VHL protein (pVHL) is widely expressed in normal human tissues, not only confounded to organs at risk for VHL disease [10,16]. In human embryos, pVHL is expressed in all three germ layers with a strong expression in the central nervous system, kidneys, testes and lungs[17]. pVHL forms a complex with different proteins including transcription factors Elongin B and C. That binding site is often affected in VHL disease. pVHL inhibits transcription elongation. The VHL protein has been shown to play an important role in the degradation of the transcription factor hypoxia-inducible factor 1α (HIF-1α). When HIF-1α is not properly degraded, an overproduction of vascular endothelial growth factor (VEGF) occurs. Cells act as if they are short of oxygen and as a result excessive blood vessel formation develops [18,19]. Both haemangioma and renal cell carcinoma show an abundance of blood vessels which is mainly driven by VEGF overproduction [20]. A strong correlation between overproduction of VEGF mRNA and hypoxia is a common characteristic in human malignancies. While haemangiomas and renal cell carcinomas are highly vascularised, the vascularisation of VHL pheochromocytomas is less prominent.

In patients with a germline mutation in the VHL gene causing its inactivation, a tumour cell can develop when the wild type allele is lost. In most VHL-disease related tumours, this "loss of heterozygosity" has been demonstrated [21]. There is a diverse collection of mutations throughout the VHL-gene. In forty percent a missense mutation is found.

In VHL disease, pheochromocytomas are solely found in type 2 caused by certain missense mutations, leading to a change in one single amino acid [22]. It is hypothesized that another tumourigenic mechanism exists for missense mutations in VHL type 2C (gain of function) as compared to missense mutations in VHL type 1 (loss of function). It is still unclear why two apparently opposing effects are caused by one and the same missense mutation, leading to different types of tumours in VHL type 2A and 2B [14,15].

From *in vivo *experiments in mice, Hoffmann *et al*., has described a group of VHL type 2C mutants which all retained the ability to interact with HIF and to down regulate HIF target genes when reintroduced into pVHL defective tumour cells. This provides a biologically conceivable mechanism to account for the low risk of haemangioblastomas associated with these mutants. Furthermore all type 2C mutants showed decreased fibronectin binding which is required for extracellular matrix formation. pVHL fulfills this function by direct binding to fibronectin.' This suggests that decreased fibronectin binding is important for pheochromocytoma development.

Gain of function in VHL2C may induce tumourigenesis of chromaffin cells directly.

It would be interesting to investigate whether this effect also plays a role in pheochromocytoma development in VHL type 2A and 2B [13-15].

In the Human Gene Mutation Database 6 previously reported mutations at the 93 codon are listed. Only one involved the Gly93Ser mutation (UMD 140) and was reported by Zbar [23]. The related phenotype is associated with a high risk of pheochromocyomas. It is unclear whether this phenotype solely involves pheochromocytomas. The other mutations are Gly93Asp.

### DNA analysis

In patients with familial pheochromocytoma screening for gene mutations in the RET, VHL, SDHB and SDHD gene is recommended since different familial syndromes can be revealed.

In one hundred percent of families with a classical presentation of more than one affected family member or a classic sporadic patient with multiple VHL-related tumours, a germline mutation is found in the VHL-gene [6,24,25]. These germline mutations are not solely found in classical families, but are also found in patients with a typical VHL tumour, lacking a positive family history. In these cases there are several possibilities. Either the family history is not sufficient or cannot be taken, or one of the parents is a carrier but has no expression of the disease, the possibility of non-paternity or it is a '*de novo*' mutation. *De novo *mutations occur in 20% of the identified germline mutation carriers.

In all patients suspected of bearing VHL disease DNA analysis is indicated. Evidence of a VHL germline mutation is necessary for confirmation of the clinical diagnosis and or presymptomatic diagnostic evaluation of family members.

### Periodical clinical evaluation

Four categories of patients with VHL disease are eligible for periodical clinical evaluation:

1) carriers of a VHL-gene germline mutation 2) first and second degree family members in a VHL family with no known germline mutation, 3) first and second degree family members of patients who decline DNA analysis and 4) patients with a typical VHL tumour without a germline mutation, but with a strong suspicion of hereditary tumours. Because of the involvement of multiple organs in VHL disease, periodical clinical evaluation should take place in a well coordinated, multidisciplinary setting. Although evidence exists for a genotype-phenotype correlation (table 1) these have limited consequences for follow-up. In VHL type 1, screening for pheochromocytomas could be less intensive than in the presented family. In type VHL 2C at least annual measurement of catecholamines and metabolites in 24 hours-excreted urine is indicated [26,27].

## Conclusion

The near fatal outcome for both mother and child as described in this report is a clear reminder of the importance of careful family history taking in hypertensive pregnant patients. The significance of genetic testing in patients presenting with bilateral pheochromocytoma is affirmed by this case with pheochromocytomas as part of VHL disease.

## Competing interests

The author(s) declare that they have no competing interests.

## Authors' contributions

JMJS studied the case and her family extensively and wrote the article

BAZ is examining the patient and her family periodically

JWM H was responsible for the molecular and pathophysiological aspects of this case

FJH takes care for the VHL-patients data base and studied the expression of the disease in families and especially in this patient

IHMBR operated on this patient and is involved in fundamental studies on VHL

CJML was responsible for the counseling of this patient and her family, and the co-ordination of the attendance.

All authors read and approved the final manuscript for publication

## References

[B1] Amar L, Bertherat J, Baudin E, Ajzenberg C, Bressac-de Paillerets B, Chabre O, Chamontin B, Delemer B, Giraud S, Murat A, Niccoli-Sire P, Richard S, Rohmer V, Sadoul JL, Strompf L, Schlumberger M, Bertagna X, Plouin PF, Jeunemaitre X, Gimenez-Roqueplo AP (2005). Genetic testing in pheochromocytoma or functional paraganglioma. J Clin Oncol.

[B2] Bravo EL, Tagle R (2003). Pheochromocytoma: state-of-the-art and future prospects. Endocr Rev.

[B3] Dahia PL (2006). Evolving concepts in pheochromocytoma and paraganglioma. Curr Opin Oncol.

[B4] Kebebew E, Duh QY (1998). Benign and malignant pheochromocytoma: diagnosis, treatment, and follow-Up. Surg Oncol Clin N Am.

[B5] Neumann HP, Bausch B, McWhinney SR, Bender BU, Gimm O, Franke G, Schipper J, Klisch J, Altehoefer C, Zerres K, Januszewicz A, Eng C, Smith WM, Munk R, Manz T, Glaesker S, Apel TW, Treier M, Reineke M, Walz MK, Hoang-Vu C, Brauckhoff M, Klein-Franke A, Klose P, Schmidt H, Maier-Woelfle M, Peczkowska M, Szmigielski C, Eng C (2002). Germ-line mutations in nonsyndromic pheochromocytoma. N Engl J Med.

[B6] Stolle C, Glenn G, Zbar B, Humphrey JS, Choyke P, Walther M, Pack S, Hurley K, Andrey C, Klausner R, Linehan WM (1998). Improved detection of germline mutations in the von Hippel-Lindau disease tumor suppressor gene. Hum Mutat.

[B7] van Heerden JA, Roland CF, Carney JA, Sheps SG, Grant CS (1990). Long-term evaluation following resection of apparently benign pheochromocytoma(s)/paraganglioma(s). World J Surg.

[B8] Whalen RK, Althausen AF, Daniels GH (1992). Extra-adrenal pheochromocytoma. J Urol.

[B9] Opocher G, Conton P, Schiavi F, Macino B, Mantero F (2005). Pheochromocytoma in von Hippel-Lindau disease and neurofibromatosis type 1. Fam Cancer.

[B10] Hes FJ, Hoppener JW, Lips CJ (2003). Clinical review 155: Pheochromocytoma in Von Hippel-Lindau disease. J Clin Endocrinol Metab.

[B11] Maher ER, Kaelin WG (1997). von Hippel-Lindau disease. Medicine (Baltimore).

[B12] Proye CA, Vix M, Jansson S, Tisell LE, Dralle H, Hiller W (1994). "The" pheochromocytoma: a benign, intra-adrenal, hypertensive, sporadic unilateral tumor. Does it exist?. World J Surg.

[B13] Hoffman MA, Ohh M, Yang H, Klco JM, Ivan M, Kaelin WG (2001). von Hippel-Lindau protein mutants linked to type 2C VHL disease preserve the ability to downregulate HIF. Hum Mol Genet.

[B14] Friedrich CA (2001). Genotype-phenotype correlation in von Hippel-Lindau syndrome. Hum Mol Genet.

[B15] Kim WY, Kaelin WG (2004). Role of VHL gene mutation in human cancer. J Clin Oncol.

[B16] MELMON KL, ROSEN SW (1964). LINDAU'S DISEASE. REVIEW OF THE LITERATURE AND STUDY OF A LARGE KINDRED. Am J Med.

[B17] Los M, Jansen GH, Kaelin WG, Lips CJ, Blijham GH, Voest EE (1996). Expression pattern of the von Hippel-Lindau protein in human tissues. Lab Invest.

[B18] Richards FM, Schofield PN, Fleming S, Maher ER (1996). Expression of the von Hippel-Lindau disease tumour suppressor gene during human embryogenesis. Hum Mol Genet.

[B19] Stebbins CE, Kaelin WG, Pavletich NP (1999). Structure of the VHL-ElonginC-ElonginB complex: implications for VHL tumor suppressor function. Science.

[B20] Mitch WE, Goldberg AL (1996). Mechanisms of muscle wasting. The role of the ubiquitin-proteasome pathway. N Engl J Med.

[B21] Kaelin WG (2002). Molecular basis of the VHL hereditary cancer syndrome. Nat Rev Cancer.

[B22] van der HE, de Krijger RR, Dinjens WN, Weeks LE, Bonjer HJ, Bruining HA, Lamberts SW, Koper JW (1998). Germline mutations in the vhl gene in patients presenting with phaeochromocytomas. Int J Cancer.

[B23] Zbar B, Kishida T, Chen F, Schmidt L, Maher ER, Richards FM, Crossey PA, Webster AR, Affara NA, Ferguson-Smith MA, Brauch H, Glavac D, Neumann HP, Tisherman S, Mulvihill JJ, Gross DJ, Shuin T, Whaley J, Seizinger B, Kley N, Olschwang S, Boisson C, Richard S, Lips CH, Lerman M, . (1996). Germline mutations in the Von Hippel-Lindau disease (VHL) gene in families from North America, Europe, and Japan. Hum Mutat.

[B24] Beroud C, Joly D, Gallou C, Staroz F, Orfanelli MT, Junien C (1998). Software and database for the analysis of mutations in the VHL gene. Nucleic Acids Res.

[B25] Gnarra JR, Tory K, Weng Y, Schmidt L, Wei MH, Li H, Latif F, Liu S, Chen F, Duh FM, . (1994). Mutations of the VHL tumour suppressor gene in renal carcinoma. Nat Genet.

[B26] Hes FJ, van der Luijt RB (2000). [Von Hippel-Lindau disease: protocols for diagnosis and periodical clinical monitoring. National Von Hippel-Lindau Disease Working Group]. Ned Tijdschr Geneeskd.

[B27] Hes FJ, van der Luijt RB, Lips CJ (2001). Clinical management of Von Hippel-Lindau (VHL) disease. Neth J Med.

